# Supplementary material for: GDF15 knockout does not substantially impact perinatal body weight or neonatal outcomes in mice
Source: bioRxiv. 2024 May 3:2024.04.30.591359. Preprint. [Version 1] doi: 10.1101/2024.04.30.591359 (PMC11092610; doi:10.1101/2024.04.30.591359)

# Supplementary Figure 1:

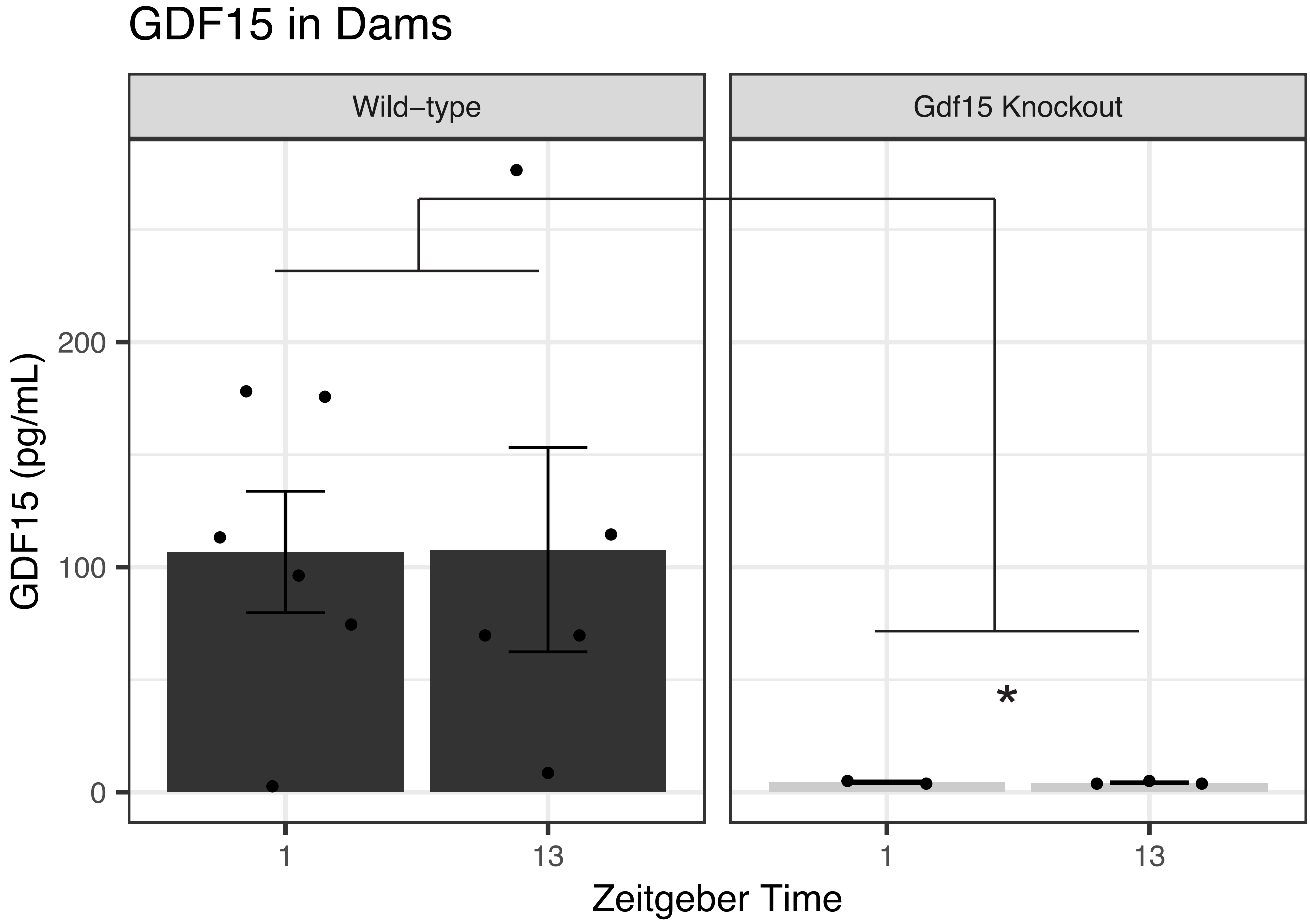

# Supplementary Figure 2:

A

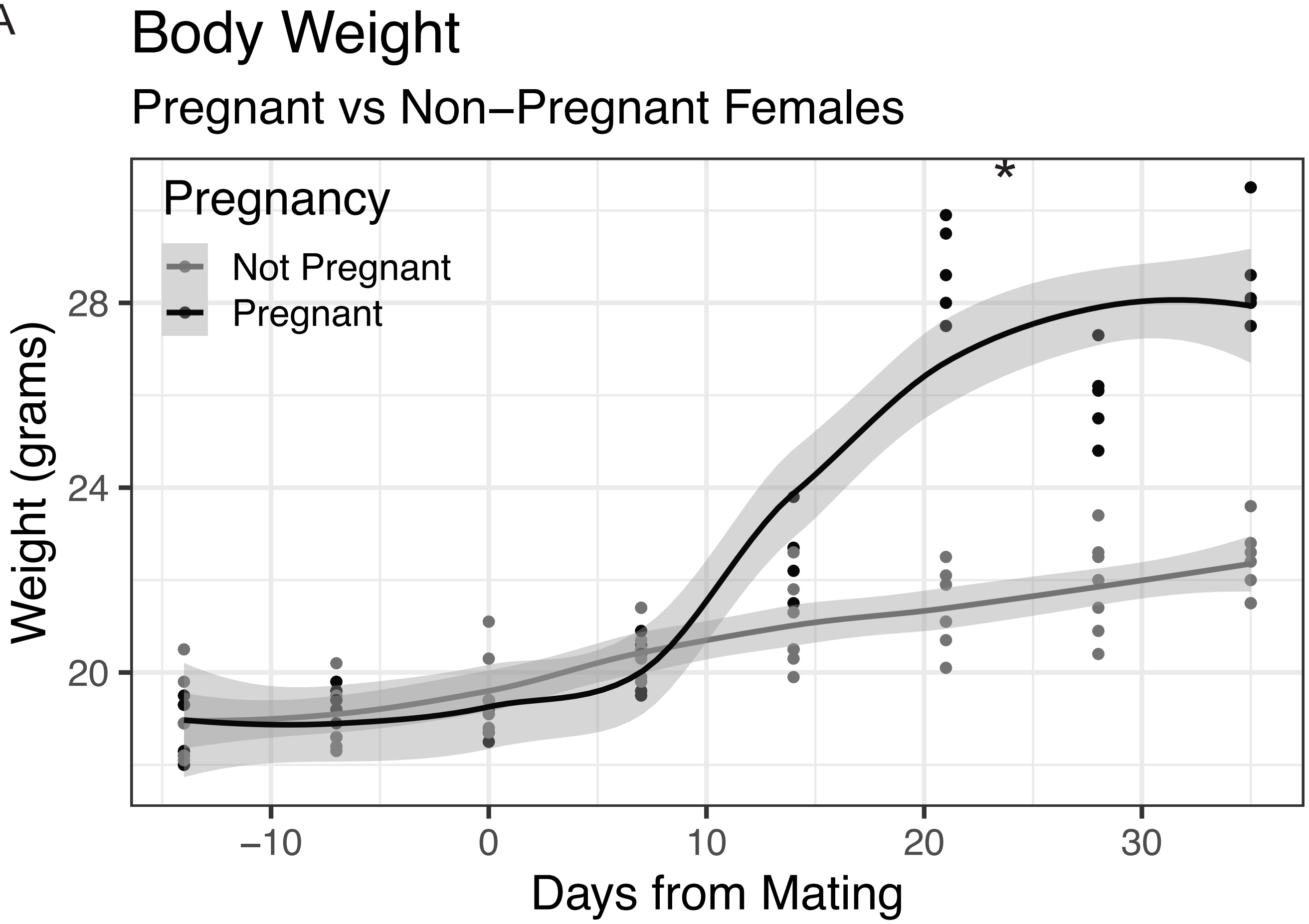


B

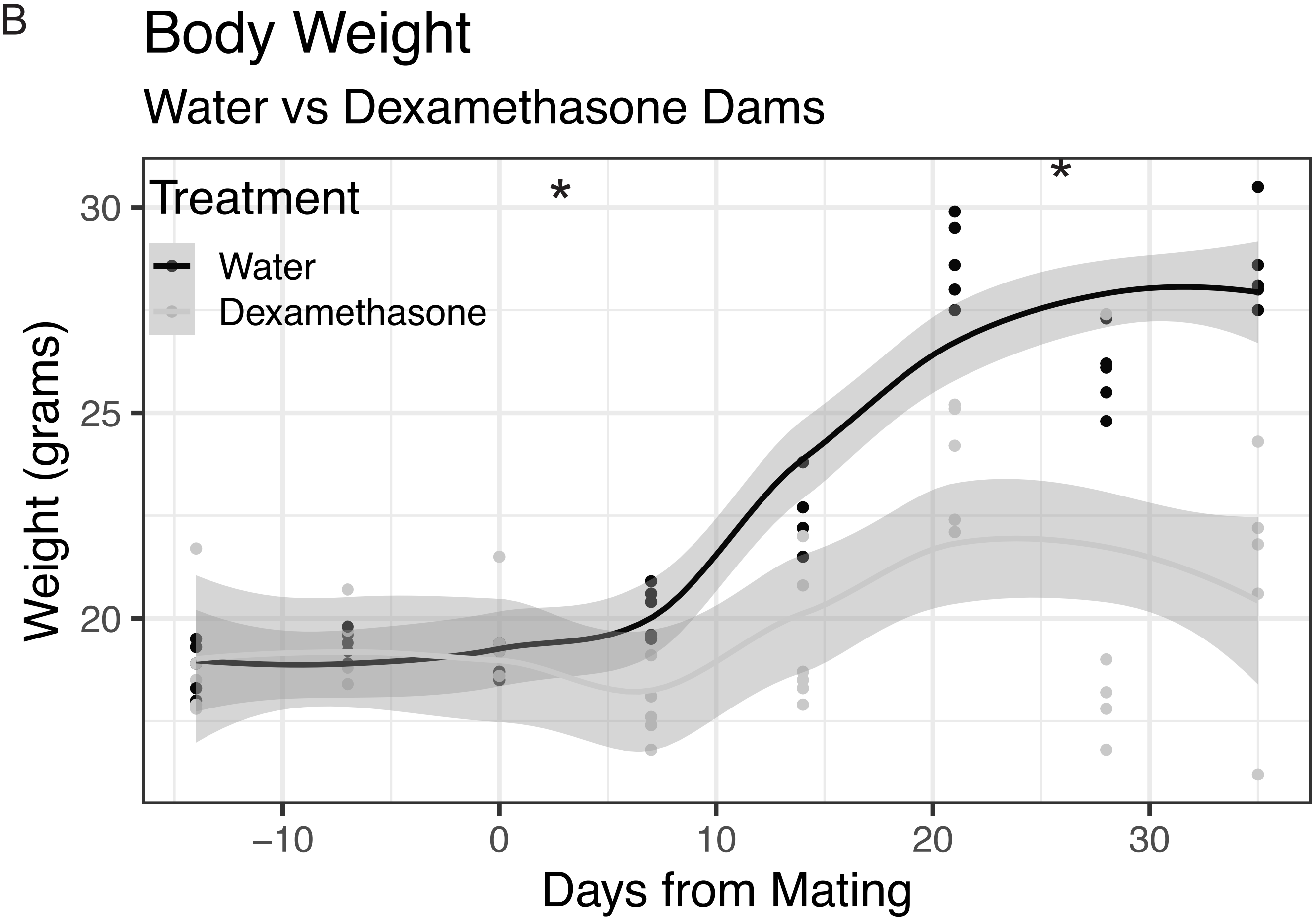

Supplement: Supplement 1 — Supplementary Figure 1: Gdf15 levels in Knockout animals and Body Weights in A) GDF15 levels in mouse serum (pg/mL) collected E16.5 at ZT1 and ZT13 in Gdf15−/− and Gdf15+/+ dams. Assessed via Students t test. * indicates p<0.05 Supplementary Figure 2: Pregnancy Increases Body Weight in Mice, but Weight Gain Is Impaired by Dexamethasone Treatment A) Body weights of non-pregnant dams compared to pregnant dams, assessed via linear mixed effect modeling. B) Body weights of pregnant dams given plain drinking water and pregnant dams given dexamethasone in drinking water, assessed via linear mixed effects modeling. * indicates p<0.05 [file media-1.pdf]
